# Phenylboronic Acids Probing Molecular Recognition against Class A and Class C β-lactamases

**DOI:** 10.3390/antibiotics8040171

**Published:** 2019-09-30

**Authors:** Pasquale Linciano, Mattia Vicario, Ivana Kekez, Pierangelo Bellio, Giuseppe Celenza, Isabel Martín-Blecua, Jesús Blázquez, Laura Cendron, Donatella Tondi

**Affiliations:** 1 Department of Life Sciences, University of Modena and Reggio Emilia, Via Campi 103, 41125 Modena, Italy; p.linciano@unimore.it; 2 Department of Biology, University of Padova, Viale G. Colombo 3, 35121 Padova, Italy; mattiavicario@hotmail.it; 3 Department of Chemistry, Faculty of Science, University of Zagreb, Horvatovac 102a, 10000 Zagreb, Croatia; ivana.pulic@gmail.com; 4 Department of Biotechnological and Applied Clinical Sciences, University of L’Aquila, via Vetoio 1, 67100 L’Aquila, Italy; pierangelo.bellio@univaq.it (P.B.); giuseppe.celenza@univaq.it (G.C.); 5 National Center of Biotechnology-CSIC, Calle Darwin 3, 28049 Madrid, Spain; imartin@cnb.csic.es (I.M.-B.); blazquez@cnb.csic.es (J.B.)

**Keywords:** Serine β-lactamases, carbapenemases, KPC-2 Klebsiella pneumoniae, GES-5 Guyana extended-spectrum-lactamase, boronic acid, enzyme inhibitors, X-ray crystallography, synergism

## Abstract

Worldwide dissemination of pathogens resistant to almost all available antibiotics represent a real problem preventing efficient treatment of infectious diseases. Among antimicrobial used in therapy, β-lactam antibiotics represent 40% thus playing a crucial role in the management of infections treatment. We report a small series of phenylboronic acids derivatives (BAs) active against class A carbapenemases KPC-2 and GES-5, and class C cephalosporinases AmpC. The inhibitory profile of our BAs against class A and C was investigated by means of molecular docking, enzyme kinetics and X-ray crystallography. We were interested in the mechanism of recognition among class A and class C to direct the design of broad serine β-Lactamases (SBLs) inhibitors. Molecular modeling calculations vs GES-5 and crystallographic studies vs AmpC reasoned, respectively, the ortho derivative **2** and the meta derivative **3** binding affinity. The ability of our BAs to protect β-lactams from BLs hydrolysis was determined in biological assays conducted against clinical strains: Fractional inhibitory concentration index (FICI) tests confirmed their ability to be synergic with β-lactams thus restoring susceptibility to meropenem. Considering the obtained results and the lack of cytotoxicity, our derivatives represent validated probe for the design of SBLs inhibitors.

## 1. Introduction

The emergence and worldwide spread of multidrug-resistant (MDR) *Enterobacteriaceae* represents a serious public health problem [1]. *Enterobacteriaceae* are common pathogens responsible for community—and hospital—acquired infections. Notably, MDR *Enterobacteriaceae* can produce β-lactamases (BLs), extended-spectrum BLs (ESBLs) and BLs carbapenemases. They inactivate the last generation of β-lactam antibiotics and the last resort carbapenems, menacing the treatment of highly resistant infections [2,3,4,5]. Therefore, antimicrobial resistance in these bacteria causes high impact on the success of treatments and reduces critically the armamentarium of antimicrobials that are still efficacious.

According to their amino acid sequences, β-lactamases are classified into class A, C, and D enzymes, which utilize serine for β-lactam hydrolysis (SBLs), and class B metalloenzymes (MBLs), which require divalent zinc ions for substrate hydrolysis [6].

In the panel of SBLs selected for our studies, we included the plasmid-mediated carbapenemases KPC-2 and GES-5 for class A [7,8,9] and the chromosomally encoded AmpC for class C [10]. Klebsiella pneumoniae carbapenemase (KPC)-producing organisms are resistant to nearly all available β-lactams antibiotics (including cefotaxime) but also to other antimicrobial classes such as fluoroquinolones and aminoglycosides [11]. GES-type β-lactamases, belonging to class A carbapenemases, have been increasingly reported among Gram-negative pathogens, including *P*. *aeruginosa*, *Enterobacter cloacae*, *Klebsiella pneumoniae*, and *Acinetobacter baumannii.* At present, 37 GES-variants have been detected worldwide [12]. AmpC β-lactamases are clinically relevant cephalosporinases. They mediate resistance to cephalothin, cefazolin, cefoxitin, most penicillins, and to β-lactamase inhibitor (BLI)-β-lactam combinations. In many bacteria, AmpC enzymes are inducible and can be over-expressed by mutation thus extending resistance to broad-spectrum cephalosporins i.e. cefotaxime, ceftazidime, and ceftriaxone [10]. In addition, AmpC can be encoded by plasmids causing its dissemination in bacteria poorly expressing the chromosomal *AmpC* gene, such as *Escherichia coli*, *Klebsiella pneumoniae*, and *Proteus mirabilis,* leading to the development of resistant bacteria overexpressing BLs belonging to class A and C [13]. In addition, in AmpC producers, the resistance is often co-mediated by mutations leading to the hyperproduction of AmpC β-lactamase, reduced influx by outer membrane porin loss and/or enhanced efflux by efflux pump activation.

At present, carbapenems are consider the first-choice drug for the treatment of infection caused by bacteria expressing multiple BLs and carbapenemases. Unfortunately, the rate of bacteria resistance to carbapenems is rising. In Enterobacteriaceae producing carbapenemases (CPE), “second line” highly toxic drugs (i.e., polymyxins, tigecycline, aminoglycosides, and fosfomycin) are often needed [14]. However bacterial strains resistant even to the second-line antibiotic treatment are growing fast and represent a pressing and challenging issue in anti-infective therapy. New alternatives are urgently needed.

**Figure 1:**
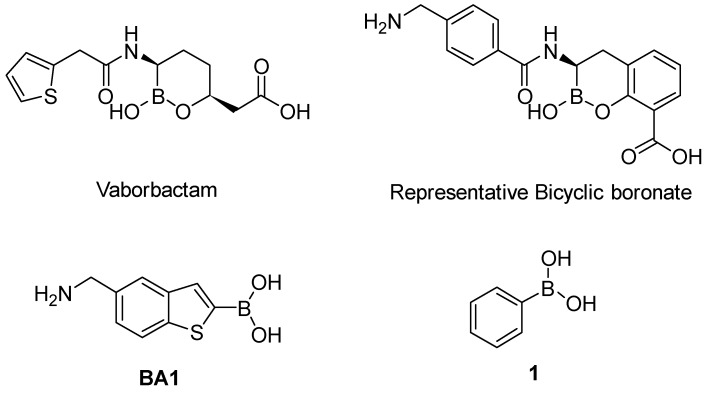
Chemical structures of Vaborbactam [16,17], a representative bicyclic boronate [18], acyclic boronic acid derivative BA1 [21,22] and phenylboronic acid 1 [24].

Among the de novo strategies directed to the inactivation of BLs, the design of covalent BLIs has represented a successful strategy to counteract resistance in pathogenic bacteria [15]. In this scenario, boronic acids (BAs) deserve a leading role and their potentiality in the design of pan-spectrum BLI has been widely demonstrated: Vaborbactam, a cyclic boronic acid active against all SBLs classes, has been recently approved in therapy [16,17] while others cyclic derivatives are now under investigation as broad spectrum, cross-classes, BLs inhibitors [18] (Figure 1).

Over the years, we have disclosed several BAs derivatives as well as other non β-lactam like moieties, active against SBLs and as broad-spectrum cross-classes BLI [19,20,21,22]. In some cases, they have shown interesting biological activity vs. clinical strains and, in light of their chemical novelty, designed molecules were able to escape pre-existing mechanism of resistance [23]. In a recent study, we reported the rational optimization of phenylboronic acid derivatives acting as novel, non-β-lactam-like, micromolar inhibitors of KPC-2 (Figure 1) [24]. The new derivatives restored susceptibility to meropenem in clinical strains overexpressing KPC-2 while cell-viability assays showed no cytotoxicity [24]. Considering these promising in vitro and in vivo results and with the intent to develop broad-spectrum SBLs compounds, in the present work we extended the study to the emergent carbapenemase GES-5 and to cephalosporinase AmpC. To determine the binding orientation of the most interesting inhibitors vs GES-5 and AmpC, molecular modeling analysis and X-ray crystallographic studies were undertaken. The obtained chemical and biological information highlighted the structural determinant guiding molecular recognition in SBLs and hence targetable in inhibitor design.

## 2. Results

### 2.1. Phenyl Boronic Acid Derivatives as Cross-classes SBLs Inhibitors

Our small library of derivatives of phenylboronic acid **1** (Figure 1), decorated in ortho or meta positions with an acrylic (**2–3**) or propionic chains (**4–5**), where assessed against class A (KPC-2 and GES-5) and class C (AmpC) SBLs. *K*_i_ values were obtained from IC_50_ as per competitive inhibition (Cheng–Prusoff equation) [25], following the inhibition patterns for this series of molecules (Table 1).

Analyzing the inhibition profile along the series, all compounds share a broad SBLs inhibitory profile with *K*_i_ in the micromolar range. Compounds **2–5** highlight the importance of the substitution position for the carboxylate moiety in modulating affinity and potency vs class A, here represented by KPC-2 and GES-5, and vs class C cephalosporinases, here represented by AmpC. Interestingly, results vs class A show ortho derivatives **2** and **4** showing higher potency vs KPC-2 and GES-5 compared to AmpC, against which the ortho substitution is detrimental for affinity (up to 50-fold drop in potency). On the contrary meta derivatives **3** and **5** resulted more active against class C with respect to class A, against which the meta substituent affects negatively the binding affinity (over 600-fold drop in potency against GES-5 and up to 47-fold against KPC-2) (Table 1).

The binding orientation of compounds **2** and **4** interacting in KPC-2 and GES-5 binding site were analyzed by X-ray crystallography and molecular modeling, respectively. The results highlighted the ability for these molecules to interact with the carboxylate-binding pocket in the active site by Arg220, Ser130, Thr237 and Thr235 in KPC-2 and by Arg244, Ser130, Thr237 and Thr235 in GES-5. For AmpC, compound **3** was selected for X-ray crystallography studies elucidating the binding potency of meta derivatives in class C.

### 2.2. Prediction of the Binding Mode of Compound 2 in GES-5 by Docking Calculation

**Figure 2:**
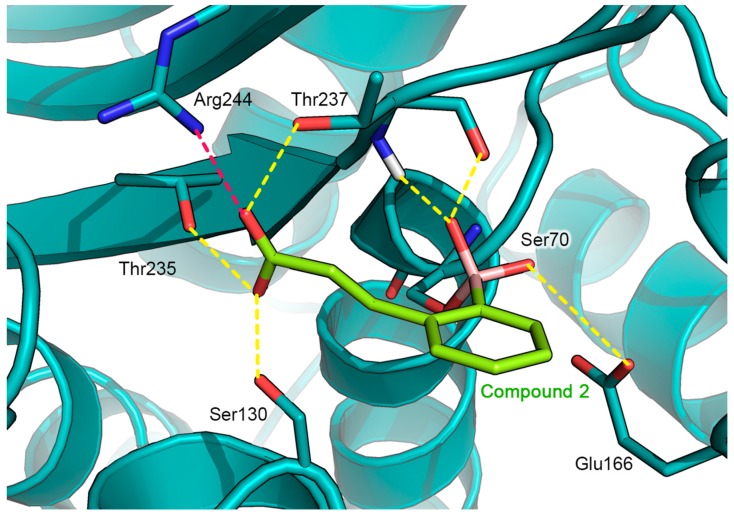
Docking pose of compound **2** (in green) as predicted in GES-5 active site (PDB ID: 6Q35, teal cartoon). Key catalytic residues are reported in deep teal sticks. H-bond are shown as yellow dashed line and the salt bridge in the magenta dashed line. Heteroatoms are color-coded (oxygen in red, nitrogen in blue, boron in pink).

The sub-micromolar binding affinity of [2-(2-carboxyvinyl)-phenyl]boronic acid (**2**) against GES-5 (*K_i_* = 0.11 μM) was investigated via covalent docking simulations. The recently disclosed X-ray structure of GES-5 in complex with a benzothiophene-2-boronic acid derivative (BA1, PDB ID: 6Q35, Figure 1) [21] was used as the reference protein for docking. Compound **2** was covalent-docked into GES-5 active site by using the CovDock utility implemented in the Schrodinger suite [26]. The catalytic hydroxyl group of Ser70 was defined as the reactive site forming a covalent bond by nucleophilic addition to the boronic acid of the inhibitor 2. The resulting predicted binding mode of **2** in complex with GES-5 is illustrated in Figure 2.

As already observed for previously resolved boronic acids-SBLs complexes, the boronic acid moiety of compound **2** represents the driving force for the covalent binding to the enzyme, sinking the inhibitor deeply in the binding site. Indeed, the boron atom of the boronate moiety is covalently bonded to the hydroxyl group of the catalytic Ser70, assuming a tetrahedral sp^3^ hybridization. In addition, the two hydroxyls groups of boronic acid establish a network of H-bonds with the surrounding amino acids of the catalytic pocket. In particular, one of the two boronic acid hydroxyls interacts with the carboxylate side chain of Glu166, whereas the second one hydrogen bonds to the backbone CO group of Thr237 and with the backbone NH group of the catalytic Ser70. According to the hybridization and geometry assumed by the boronic acid, the inhibitor adopts a de-acylation transition-state analog conformation [24]. Indeed, one boron oxygen is located in the oxyanion hole, whereas the other displaces the de-acylation water normally positioned between Glu166 and Ser170.

**Figure 3:**
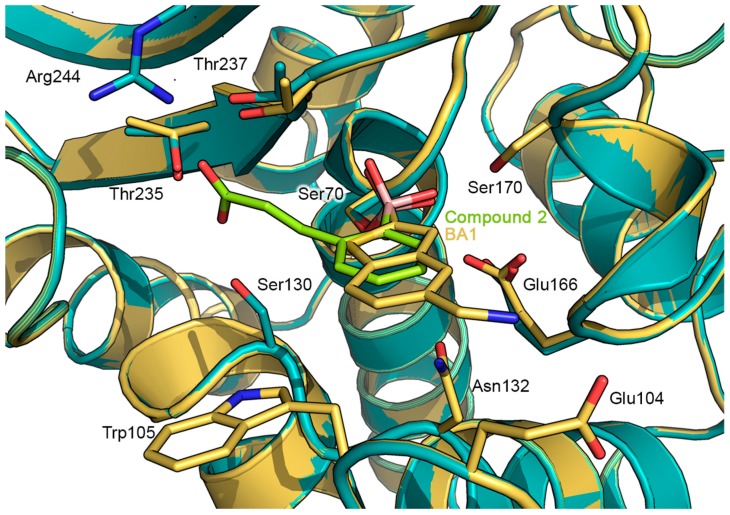
Superimposition of the predicted docking pose of compound **2** (green sticks) in complex with GES-5 protein (PDB ID: 6Q35, deep teal cartoon), with the X-ray structure of benzothiophene-2-boronic acid (**BA1**) (yellow/orange sticks) in complex with GES-5 (PDB ID: 6Q35, pale yellow cartoon) [21]. Important amino acidic residues are shown as sticks, by using the color code adopted for the respective proteins. Heteroatoms are color-coded (oxygen in red, nitrogen in blue, boron in pink).

Comparable to the binding pose of BA1, the phenyl ring of compound **2** is located perpendicularly to the surface of the catalytic binding pocket, pointing toward the solvent-exposed entrance of the cleft. However, the smaller aromatic moiety of compound **2** is not able to establish hydrophobic interactions with the aminoacidic residues of the cleft, as observed for the larger benzo[b]thiophene ring of compound BA1 (Figure 3) [21].

**Figure 4:**
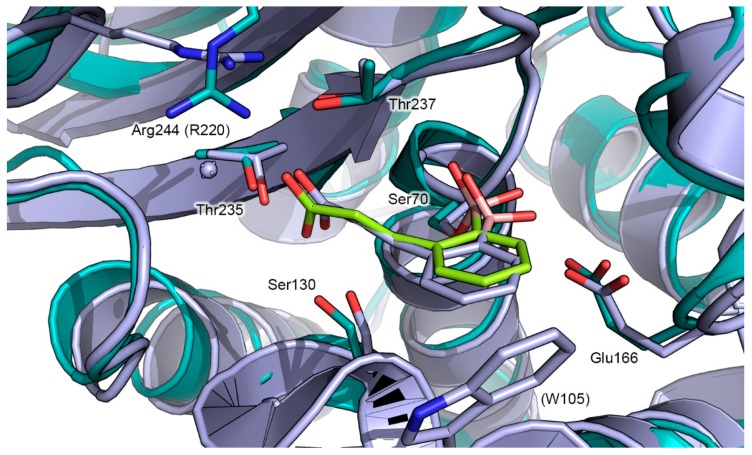
Superimposition of the predicted docking pose of compound **2** (green sticks) in complex with GES-5 protein (PDB ID: 6Q35, teal cartoon), with the X-ray structure of **2** (indigo sticks) in complex with KPC-2 (PDB ID: 5LL7, indigo cartoon) [24]. Residues involved in binding are shown as sticks, using the color code adopted for the respective proteins. The corresponding amino acidic numeration for KPC-2 is reported in parenthesis. Heteroatoms are color-coded (oxygen in red, nitrogen in blue, boron in pink).

Finally, compound **2** orients the ortho carboxyvinyl lateral chain toward the C3(4’) carboxylate binding pocket, highly conserved in all SBLs, similarly to the binding mode adopted by compound **2** in complex with KPC-2. The carboxylate group is involved in a pattern of hydrogen bonds with the side chain hydroxyls of Ser130, Thr235 and Thr237. Interestingly, in GES-5, the carboxylate group seems to be involved in a strong salt bridge with the positively charged guanidine side chain of Arg244 located at an average distance of 2.45 Å. In contrast, in KPC-2 in which Arg244 is not conserved, the above described specific interaction could be picked up by Arg220, oriented in the proximity of the lateral chain of **2**. However, Arg220 in KPC-2 appears oriented in a more distant position respect to that adopted by Arg244 in GES-5, and an ordered water molecule mediates a specific H-bond interaction with the carboxylic group of compound 2 (Figure 4). The lack of the salt bridge in KPC-2 justifies the 23-fold higher activity of **2** against GES-5 (*K*i of 0.108 µM) with respect to KPC-2 (*K*i of 2.43 µM).

Comparing the predicted GES-5: compound **2** complex with the X-ray structure of GES-5:BA1 (Figure 3) [21] and the X-ray structure of KPC-2: Compound **2** (PDB ID: 5LL7, Figure 4) [24], close analogies between binding modes emerged. The binding mode adopted by compound **2** in GES-5 active site closely resembles that of BA1, with the boronic moiety and the aromatic part of the ligand engaged in similar interactions [21]. Moreover, compound 2 maintains a binding mode similar to that determined in the previously released X-ray KPC-2: **2**: in particular, the later carboxylated chain is engaged in an extended network of specific interactions, involving Arg244, Thr237, and 235 and Ser130 [24].

### 2.3. AmpC-Compound 3 Binary Complex

The structure of the AmpC:**3** complex was solved at 1.78 Å resolution, providing a detailed picture of compound **3** binding orientation in AmpC active site (statistics data are reported in Table S1). The asymmetric unit contains one protein molecule, 131 water molecules, one molecule of **3** and one phosphate ion. The electron density for residues Gly1, Glu2, Lys370, and Arg371 was not observed and was not included in the model. The final model was refined to a *R*_work_ = 17.7% and a *R*_free_ = 20.0%. Clear electron density in the difference maps was observed in the active site allowing proper fitting of the inhibitor molecule and determination of any rearrangements in the pocket upon binding.

**Figure 5:**
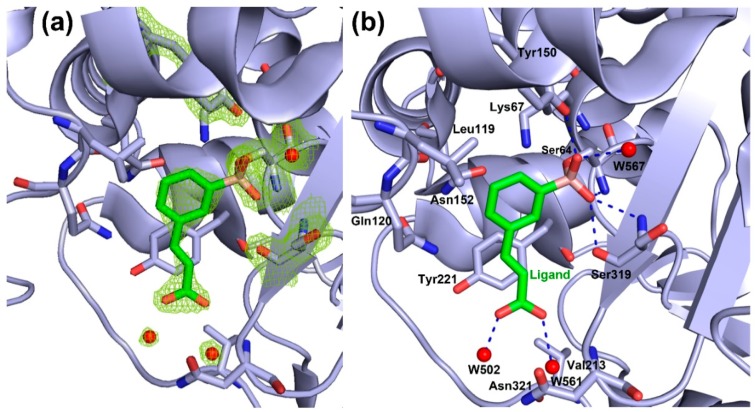
Binary complex of *Pa*AmpC:**3**. (**a**) The 2*F*o-*F*c electron density map of the active-site residues contoured at 2.0 σ level is shown in green mesh. (**b**) Residues around the binding pocket of the *Pa*AmpC:**3** crystal structure involved in the main interactions with the inhibitor molecule are shown as sticks (carbon atoms in green for the ligand and in light blue for *Pa*AmpC, oxygen atoms are in red, nitrogen atoms in blue and the boron atom in pink). Polar interactions within 3 Å are shown in blue dashed lines. Water molecules interacting with the ligand molecule are depicted as a red sphere. Pictures were prepared by using PyMOL [29].

**Figure 6:**
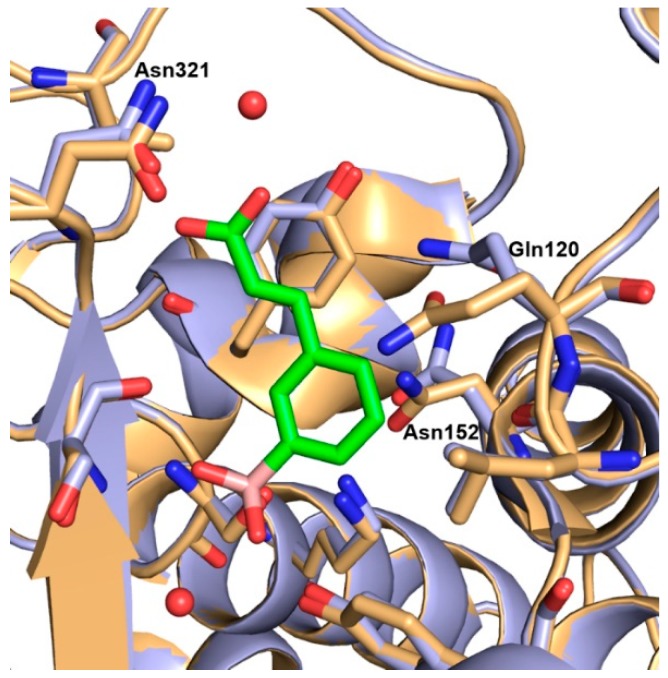
Overlay of the native *Pa*AmpC active site (light orange, PDB ID: 4GZB [30]) on *Pa*AmpC (light blue) covalently bound to **3** (green). The residues surrounding the ligand are shown as sticks. Sidechains of the residues showing the major structural change are labeled. Water molecules from the *Pa*AmpC:**3** structure are shown as red spheres.

Inhibitor **3** is covalently bound through the boron atom to the side chain oxygen atom (O_γ_) of Ser64 (Figure 5A,B and Figure S1), adopting a tetrahedral geometry as already reported for other similar complexes [27,28]. The catalytic and conserved residues in AmpC β-lactamase catalytic domain undergo minimal rearrangements upon binding (Figure 6).

Neighboring boronate oxygen atom O14 makes hydrogen bonds with the backbone NH of catalytic Ser64 and with the backbone NH of Ser319. The boronic acid oxygen atom O13 forms strong hydrogen bonds with the −OH group of the side chain Tyr150 and with a water molecule W567, commonly highly conserved in AmpC binary complexes with aryl boronic acid inhibitors [27]. The meta carboxyvinyl lateral chain of the ligand molecule is placed near Tyr221 and is stabilized by the π⋅⋅⋅H−C interaction. The carboxylate group of the meta carboxyvinyl lateral chain is orientated toward the amide group of Asn321 and the methyl group of Val213. In class C SBLs, the carboxylate group does not interact in the typical carboxylate site of antibiotics as in class A SBLs, but, on the contrary, is oriented in the opposite direction, towards Asn152, Gln120, and Leu119, where usually the R1 chain of antibiotics binds [27]. The carboxylate group of the inhibitor **3** is stabilized through the hydrogen bonding of the oxygen atoms O01 and O03 and water molecules W561 and W502, respectively. Moreover, these water molecules make a continuous network of hydrogen bonds with the backbone NH of Asn321 and methyl group of Val213 (Figure S1, for hydrogen bond distances refer to Table S2).

The orientation of the aromatic moiety in the ligand molecule is toward the Asn152, Lys67 and Leu119 and these residues form C=O⋅⋅⋅π, N−H⋅⋅⋅π, and O⋅⋅⋅H−C interactions, respectively, with the phenyl ring. Moreover, the aromatic moiety is surrounded by residues Gln120 and Asn152 defining the opening of the binding site.

The *Pa*AmpC:**3** monomer superimposes well with the monomer of the native *Pa*AmpC (PDB ID: 4GZB [30]). The major structural change involves the region near the phenyl ring of compound **3** and comprises a conformational shift of the side chain residues Gln120 and Asn152 (Figure 6). These residues are important in the substrate selectivity in class C BLs and play an important role in kinetics of the substrate hydrolysis. Comparing to the structure of native *Pa*AmpC, in the crystal structure of *Pa*AmpC: **3** the side chain of Gln120 is rotated by 50 degrees and the side chain of the Asn152 accommodates a flipped orientation and show a shift of 36 degrees. Interestingly, in the crystal structure of *Pa*AmpC bound to the inhibitor avibactam (PDB ID: 4HEF [30]) these residues superpose well with the native *Pa*AmpC structure while the largest change is found in the orientation of the Tyr150 sidechain where avibactam binding causes planar rotation by 50 degrees of the Tyr150 ring [30]. This finding suggests that, depending on the size and bulkiness of the inhibitors, the proximal residues are prone to arrange to better allocate encumbering structures.

Comparing the complex *Pa*AmpC:**3** with that of compound **2** bound to the KPC-2 protein [24], significant differences in the binding orientation emerged. In KPC-2 the ligand molecule orients its ortho carboxyvinyl lateral chain towards the carboxylate binding pocket forming a network of hydrogen bonds with the Thr237, Thr235, Ser130 sidechains and a highly conserved water molecule (Figure 2). In contrast, in the *Pa*AmpC:**3** complex the inhibitor points its *meta* carboxyvinyl group towards the opposite direction, extensively interacting with the aqueous solvent (Figure 5B and Figure S1).

As the matter of fact, in AmpC the lateral carboxylated side chain of **3** does not reach the oxyanion hole, as for compounds **2** and **4** in class A, being oriented in the opposite direction, thus losing the possibility to contact key active site residues. No strong specific interactions resulted for the lateral carboxylated chains, as well as no unfavorable clashes with the protein. This accounts for the similar binding potency of compounds **3** (*K*_i_ 1.45 μM) and **5** (*K*_i_ 5.3 μM) with respect to the lead phenylboronic acid **1** (*K*i 4.85 μM) [24].

### 2.4. Biological Evaluation Against Clinical Strains

To investigate the ability of our phenylboronic acid derivatives **2**-**5** to reach the periplasmic space, where SBLs are secreted and concentrated in Gram negative bacteria, and synergically protect β-lactam antibiotics hydrolysis by BLs, drug interactions model *via* the fractional inhibitory concentration index (FICI) was determined against clinical strains overexpressing BLs targets of our studies (Table 2). FICI of meropenem (MEM) or ceftazidime (CAZ) plus inhibitors against clinical isolates of *Klebsiella pneumoniae* expressing KPC-2 and *Pseudomonas aeruginosa* overexpressing AmpC were employed.

Results from drug–drug interactions assay are consistent with those obtained from the in vitro studies (Table 1) and the preference for the class of enzymes is maintained in biological tests (Table 2). Indeed, our phenylboronic acids act synergistically on the base of their preference for class A or class C enzymes. As far as potency is concerned, compounds **2** and **4** exert a clear synergistic activity towards *Klebsiella pneumoniae* strains in combination with MEM, with FICI values well below the cut-off value of 0.5. Instead, in *Pseudomonas aeruginosa* compounds **3** and **5**, although synergism can be defined in combination with CAZ, their FICI values are around the cut-off value, with the exception of the combination CAZ: **5** against *P. aeruginosa* strain 148.

## 3. Discussion and Conclusions

A small set of phenylboronic acids were identified as low micromolar inhibitors of SBLs belonging to class A (KPC-2 and GES-5) and C (AmpC). In biological tests conducted against clinical strains, BAs derivatives were able to synergically protect meropenem from BLs hydrolysis. 

As already observed for KPC-2 inhibition, the derivatization of the phenylboronic acid **1** with the introduction in ortho position of a carboxylated chain strongly improved affinity vs KCP-2 (**2** and **4**) while being detrimental against AmpC. Interestingly, analyzing the activity of the meta derivatives **3** and **5**, we noticed a drop in affinity against class A confirming the importance of the carboxylate moiety orientation to pick specific interactions in KPC-2 and GES-5 binding pockets. The improved affinity for **2** and **4** against KPC-2 and GES-5 lies in specific interactions in the β-lactam carboxylate binding pocket [31].

Compounds **3** and **5** do not experience improvement in affinity compared to lead phenylboronic acid **1**, thus reflecting the lack of strong additional specific interactions for meta derivatives in AmpC binding site [31]. The carboxylated chain in these compounds, despite the possibility to be accommodated in the catalytic site, cannot reach key recognition binding sites like compounds **2** and **4** in KPC-2 and GES-5, thus resulting in a minimal improvement in activity against class C. On the contrary, the consistent drop in affinity of the ortho derivatives **2** and **4** against AmpC is probably related to non-favorable interaction in the active site. As a matter of fact, compounds **2** and **4**, though micromolar inhibitors, due to their ortho derivatization, cannot properly fit in the active site of *Pa*AmpC. The steric hindrance caused by the orientation of Ser319 and Thr320 in the *Pa*AmpC catalytic pocket probably disallows a proficuous accommodation of the ortho derivatives.

Interactions targetable in the design of potent phenyl BAs as SBL inhibitors can be derived by comparing the structure of AmpC in complex with ceftazidime, a class of β-lactam antibiotics, and *Pa*Ampc**:3** structure. In the structure of *Ec*AmpC bound to ceftazidime (PDB ID:1IEL [32]) the carbonyl oxygen atom O9 of the opened lactam ring establishes the same N-H⋅⋅⋅O interactions with the main chain nitrogen atoms of catalytic Ser64 and Ala318 (corresponding to Ser319 in *Pa*Ampc:**3**) as the oxygen O14 atom in the *Pa*Ampc: **3** structure. Moreover, the R1 side chain of ceftazidime is stabilized through the hydrogen bonding of the carbonyl oxygen of the R1 amide group (O12) and Asn152 and with the π⋅⋅⋅π interaction of the thiazole ring and Tyr221 and adopts the same orientation of the carboxyvinyl lateral chain of compound **3**. The mentioned residues are also important in positioning **3** in the binding pocket of *Pa*AmpC where C=O⋅⋅⋅π (between Asn152 and phenyl ring of **3**) and π⋅⋅⋅H−C (Tyr221 and the carboxyvinyl chain of **3**) interactions are involved in the stabilization of the complex structure. With respect to compound **3**, the R1 side chain of the ceftazidime molecule carries a flexible carboxylate moiety oriented toward the bulk solvent (Figure S2). In both analyzed structures, the deacetylating water molecule is present—in the *Ec*AmpC: ceftazidime structure the nitrogen atom N5 of the opened lactam ring is hydrogen-bonded with the water molecule (W402) while in the *Pa*Ampc: **3** structure the same interaction is found between the boronic acid oxygen atom O13 and the water molecule W567.

Against KPC-2, instead, the ortho derivatives **2** and **4** resulted very active since the acrylic acid side-chain orients toward the highly conserved carboxylate binding site formed by Thr235, Gly236, Thr237, and Ser130 similarly to the structure of KPC-2 bound to the β-lactam cefotaxime (PDB ID:5UJ3 [33]). The existence of the interaction between Trp105 and the phenyl ring of the compound **2** in KPC-2:**2** and between Trp105 and the six-membered dihydrothiazine group in KPC-2: cefotaxime is a consequence of a higher degree of freedom of the Trp105 side chain, prone to adopt the proper conformation depending on the substrate arrangement (Figure S3).

Similarly to KPC-2, GES-5 is strongly inhibited by the ortho derivatives **2** and **4.** The docked structure of compound **2** in GES-5 active site showed a similar conserved orientation of the carboxylated side chain as in the structure of the KPC-2:**2** complex and like the carboxylate group of the β-lactam antibiotic in the structure of GES-5 bound to imipenem (PDB ID:pymol [34]) (Figure S4). The C_3_ carboxylate part of the imipenem molecule is trapped in the carboxylate site and shows a typical pattern of hydrogen bond interactions mediated through the side chains of residues Ser130, Thr235, Thr237, Arg244, and a highly conserved water molecule. The key interactions for housing the oxyanion hole are found for both GES-5: ligand structures (main-chain N atoms of Ser70 and Thr237 with the ester carbonyl in GES-5: imipenem and backbone CO group of Thr232 and backbone NH group of the catalytic Ser64 with one of the boronic acid hydroxyls in GES-5:**2**) (Figure S4). The hydroxyethyl moiety of imipenem, complementary to the other boronic acid hydroxyl in GES-5:**2,** interacts with the Glu166 side chain (corresponds to Glu161 in GES-5:**2**) and additionally with the side chain of Asn132. Interestingly, in the docked GES-5:**2** structure Glu166 hydrogen bonds to Ser70 which is a rare case in the class A BLs. Smith et al. (2012) [34] disclosed the importance of this H-bond between the Glu166 and Ser70 side chains in the GES-5: imipenem complex, suggesting it could play a pivotal role for the carbapenemase activity. Finally, the existence of the salt bridge between the Arg244 and the carboxylate group in the GES-5: imipenem structure, as in the docked GES-5:**2** structure, contributes to the higher affinity of the compound **2** against GES-5 than against KPC-2, highlighting the importance of this particular interaction in the specific inhibitor design targeting class A SBLs.

Antimicrobial assays undertaken against five clinical strains overexpressing the BLs targets of our study highlighted the ability for our derivatives to synergize β-lactam antibiotics such as CAZ and last resort meropenem.

Our small boronic acid derivatives have clear potential as cross-class SBLs inhibitors and the available structures of KPC-2 in complex with compounds **2** and **4**, and that of **3** in complex with AmpC represent a valuable point for future hit-to-lead optimization efforts. Our results highlight the importance of contacting critical subsite in the catalytic pocket thus opening new perspectives in the drug design of improved phenylboronic acid broad spectrum SBL inhibitors targeting key and common spots of SBLs binding site.

## 4. Materials and Methods 

### 4.1. Chemistry

Compounds **2–5** (Table 1) were prepared as previously reported [24].

### 4.2. Proteins Production and Purification

Expression and purification of recombinant KPC-2 and GES-5 have been performed as already reported [21,24]. For recombinant AmpC from *Pseudomonas aeruginosa*, the optimized full-length synthetic gene was purchased (GeneArt service under Life Technologie) and subcloned into pRham vector to have it in the full-length version. Protein was expressed according to standard protocol: *P. aeruginosa* AmpC-β-lactamase was obtained from a culture of *E. coli* BL21(DE3), carrying the plasmid vector pRham-AmpC. 1L of LB-medium with kanamycin 50 μg/mL, at 37 °C, 150 RPM for 24 hrs. Expression was induced by 0.2% *w/v* Rhamnose. After 5–6 hours the cell broth was centrifuged at 4500 RPM, 4 °C, for 20 min. The sample was loaded on a column packed with 25 mL of Affigel-10 (Biorad) functionalized with 3 MAPB (3-methylaminophenylboronic acid). The column was previously equilibrated with the loading buffer. The protein was eluted (flow rate, 1 mL/min) with 5 column-volumes of elution-buffer (0.5 M boric acid, 0.5 M NaCl, pH 7.0). β-lactamase activity was checked in each elution fraction using 200 μM CENTA [35]. Active fractions were pooled, concentrated by ultrafiltration using a Millipore Ultra-15 (Ultracel-10K; Millipore) and checked by SDS-Page. The protein pool final concentration (1 mg/mL) was assessed using Bradford and UV-Vis assays. Purified AmpC β-lactamase was stored at −80 °C [36].

### 4.3. In Vitro Enzyme Inhibition Assays Against KPC-2, GES-5, and AmpC 

The half-maximal inhibitory concentration (IC_50_) of phenylboronic acid derivatives was determined as follows. Reactions were monitored using a Jasco V-730 spectrophotometer at 485 nm wavelength. Boronic acids were dissolved in dimethyl sulfoxide (DMSO) to a concentration of 10 mM and stored at −20°C. Each compound was tested at concentrations ranging from 1 µM to 200 µM for inhibitory activity vs full-length KPC-2 enzyme, GES-5 and AmpC in 50 mM of PB + 50 mM KCl at pH 7.4 at 25°C with 0.01% *v/v* Triton X-100, to avoid compound aggregation and promiscuous inhibition [37] in presence of nitrocefin as reported substrate. For tests against KPC-2, reporter substrate nitrocefin was used at 40 μM, (K_m_ 36 μM) while for tests against GES-5 and AmpC reporter substrate nitrocefin was used at 200 μM (K_m_ 208 μM and K_m_ 200 respectively). All the experiments were performed in triplicate. Experimental error never exceeded 5%. The reaction was typically initiated by adding the enzyme to the reaction buffer last. The IC_50_ values were determined by measuring the rate of hydrolysis of a reporter substrate in the presence of five different inhibitor concentrations at λ 482 nm. The binding affinity *k*_i_ was estimated, for each compound in the library, from the determined IC_50_ by Cheng–Prusoff equation as per competitive inhibition (Table 1) [25].

### 4.4. Molecular Modeling

#### 4.4.1. Proteins and Ligands Preparations

The crystal structure of GES-5, covalent bounded to benzothiophene-2-boronic acid inhibitor (PDB ID: 6Q35) was used as a reference protein for docking calculation [21]. The structure was prepared for calculation by using the Protein Preparation Wizard utilities implemented in Maestro 11.1 of the Schrodinger suite 2017-1 [38,39]. Epik was used to generate the protonation and tautomeric states of the residues at pH 7.0 ± 2.0. Water molecules, ions and co-crystallized organic solvent molecules (i.e., DMSO, glycerol ethyne glycol) were removed. The hydrogens position and H-bonds were minimized with the OPLS_2005 force field. The minimized protein was prepared for covalent docking. The B-O bond between co-crystallized ligand and Ser70 was removed; the hydrogen on the hydroxyl group of the reactive Ser70 was added back and the co-crystalized ligand removed. The chemical structure of compound 2 was drawn using ChemBio3D 14.0 and prepared using the LigPrep module of Schrödinger suite with the OPLS_2005 force field [40,41]. The prepared protein and the prepared ligand were merged into one entry in preparation for docking.

#### 4.4.2. Docking Calculation

Covalent docking calculations were performed on the prepared protein structures by using the CovDock utility implemented in Maestro 11.1 [26]. Covalent Docking in Maestro was performed by a combination of Glide and Prime programs [26]. Glide was used to dock the small molecule and Prime was used to run local sampling once the bond has formed between the protein and the ligand. The hydroxyl group of Ser70 was defined as the reactive site to form a covalent bond to the boronic acid of the designed inhibitor, as preset by CovDock (Reaction type: boronic acid addition). The pose prediction protocol was used. No position constraints were applied. Default parameters were employed for the refinement of docking poses. A maximum of 10 poses per ligand were kept and subjected to post docking minimization. Docking results were ranked according to their Prime energy and Glide score. Structural visualization and representation were performed with PyMOL [29].

### 4.5. Co-Crystallization and Structure Determination

Crystallization of *Pseudomonas aeruginosa* AmpC in complex with compound 3 ([3-(2-carboxyvinyl)phenyl] boronic acid]) (*Pa*AmpC:3) was carried out by the hanging drop vapor diffusion method. Protein sample was concentrated by ultrafiltration to 4 mg/mL in 20 mM NaH_2_PO_4_ pH 7.5, 30 mM NaCl and equilibrated over 1.8 M potassium phosphate buffer pH 8.7. The drop was prepared by mixing the protein and precipitant solutions containing the inhibitor compound (1mM AA647, 2% DMSO, 1.8 M potassium phosphate buffer pH 8.7) in equal volumes. Crystals appeared after 5 days of equilibration at 294 K. For data collection, crystals were left to equilibrate in a cryoprotectant solution containing 20% sucrose, 1.8 M potassium phosphate pH 8.7 and were flash cooled in liquid nitrogen. Data collection was performed at the ESRF BEAMLINE ID23-1 (Grenoble, France) and *Pa*AmpC:3 crystals gave diffraction data to 1.78 Å resolution. The measured data were processed by XDS [42] and then solved by molecular replacement with Phenix [43] using the structure of native *Pa*AmpC as the search model (PDB ID: 4GZB [30]). The resulting model was alternately refined with Refmac5 [44] implemented in CCP4i and then checked and manually adjusted with Coot [45]. Manual rebuilding and placement of water molecules was performed with the program Coot as well. The inhibitor molecule was built and optimized with Elbow [46], manually fitted in the difference maps by using Coot molecular graphics tools and further refined using Refmac5. LigPlot was used to analyze and visualize protein-ligand interactions [47] and MolProbity [48] was used for the refinement quality evaluation. The *Pa*AmpC:3 structure is deposited in PDB as 6S1S (PDB ID).

### 4.6. Drug Interaction Models Via Checkerboard Microdilution Assay

The in vitro interactions between the antibiotics (meropenem or ceftazidime) used for the double-disk assay and the boronic acid compounds were investigated by a two-dimensional checkerboard microdilution assay in microtiter plates, as already described [24]. To assess the nature of the in vitro interactions between the compounds and antibiotics against selected clinical strains, the data obtained from the checkerboard assay were used to calculate the fractional inhibitory concentration index (FICI) [24]. Briefly, exponential cultures of the bacterial strains in Mueller Hinton broth were inoculated in the wells of microtiter plates containing different concentrations of the cited antibiotics, with and without phenylboronic acids, and incubated at 37 °C for 18 hours. The growth in each well was quantified spectrophotometrically at 595 nm by a microplate reader TECAN Infinite (Tecan Trading AG, Switzerland). The minimal inhibitory concentration (MIC) for each drug and combination of drugs was defined as the concentration of drug that reduced growth by 80% compared to that of organisms grown in the absence of drug. The fractional inhibitory concentration index (FICI) is the mathematical expression of the effect of the combination of antibacterial agents expressed in Equation (1):

(1) $$\Sigma FIC=FIC_{A}+FIC_{B}=\frac{MIC_{AB}}{MIC_{A}}+\frac{MIC_{BA}}{MIC_{B}}$$

in which MICA and MICB are the MICs of drugs A and B acting alone, and MICAB and MICBA are the MICs of drugs A and B in combination. Synergy is defined when the FICI is ≤0.5, antagonism when the FICI is > 4, and indifference when the FICI is >0.5 and ≤4.

## Figures and Tables

**Table 1:** Inhibitory profile of phenylboronic acids against Serine *β*-Lactamases of Class A and C.

Code	Structure	KPC-2 ^[1]^ *K_i_* μM	GES-5 ^[2]^ *K_i_* μM	AmpC ^[3]^ *K_i_* μM
**2**	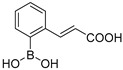	2.43	0.11	41.7
**3**	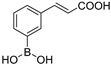	31.2	24.6	1.45
**4**	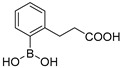	1.13	1.83	76.1
**5**	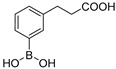	53.0	69.4	5.3

^[1]^ Estimated from IC_50_ by Cheng–Prusoff equation for competitive inhibition [25]. Reporter substrate was nitrocefin (40 μM, *K_m_* 36 μM) [24]. ^[2]^ Estimated from IC_50_ by Cheng–Prusoff equation. Reporter substrate was nitrocefin (200 μM, *K_m_* 208 μM). ^[3]^ Estimated from IC_50_ by Cheng–Prusoff equation. Reporter substrate was nitrocefin (200 μM, *K_m_* 200 μM). All experiments were performed in triplicate. Experimental error never exceeded 5%.

**Table 2:** In vitro interaction between antibiotics and boronic compounds determined by fractional inhibitory concentration index (FICI).

Checkerboard Microdilution Assays and Drug Interaction Model						
Strains	MIC ^[a]^ Antibiotic		FICI ^[b]^ Antibiotic + inhibitor			
	MEM	CAZ	2	3	4	5
Klebsiella 99D8	128	-	0.0156	2	0.1250	2
Klebsiella 53A8	128	-	0.0117	2	0.1875	2
Klebsiella 53A9	128	-	0.0175	1.5	0.2500	1.5
P aeruginosa 12	-	32	2	0.5	2	0.3750
P aeruginosa 148	-	128	1	0.375	1.5	0.0936

^[a]^ Minimal Inhibitory Concentration (μg/mL). ^[b]^ Synergy is defined when the FICI is ≤0.5, antagonism when the FICI is >4, and indifference when the FICI is >0.5 and ≤4.

## Supplementary Materials

The following are available online at https://www.mdpi.com/2079-6382/8/4/171/s1, free of charge. Table S1: Statistics of data collection, processing and refinement, Table S2: Hydrogen bonds between ligand molecule 3, PaAmpC residues and water molecules, Figure S1: Key interactions *PA*AmpC:**3**, Figure S2. Overlay of the EcAmpC: ceftazidime and PaAmpC: **3** active sites, Figure S3. Overlay of the KPC-2: cefotaxime and KPC-2:2 active sites, Figure S4. Overlay of GES-5: **i**mipenem and GES-5:**2** active sites.

## Accession Code

Atomic coordinates and structure factors have been deposited at The Protein Data Bank for immediate release as PDB ID: 6S1S.

## Abbreviations

BLs: β-lactamases; cdp: compound; CREs: Carbapenem-resistant Enterobacteriaceae, CAZ: ceftazidime; FICI: fractional inhibitory concentration index; GES-5: Guyana extended-spectrum-lactamase; KPC-2: Klebsiella pneumoniae carbapenemase 2, MDR: Multi drug resistant, MIC: minimum inhibitory concentration, MEM: meropenem; SBLs: serine β-lactamases.
