# Supplementary material for: Phenylboronic Acids Probing Molecular Recognition against Class A and Class C β-lactamases
Source: Antibiotics (Basel). 2019 Sep 30;8(4):171. doi: 10.3390/antibiotics8040171 (PMC6963673; doi:10.3390/antibiotics8040171)
Supplement: Supplementary file 1 [file antibiotics-08-00171-s001.pdf]

Supplementary Material

Phenylboronic Acids Probing Molecular Recognition against Class A and class C β-lactamases

Pasquale Linciano [1,†], Mattia Vicario [2,†], Ivana Kekez [3], Pierangelo Bellio [4], Giuseppe Celenza [4], Isabel Martín-Blecua [5], Jesús Blázquez [5], Laura Cendron [2,*] and Donatella Tondi [1,*]

1 Department of Life Sciences, University of Modena and Reggio Emilia, Via Campi 103, Modena41125, Italy
2 Department of Biology, University of Padova, Viale G. Colombo 3, Padova 35121, Italy
3 Department of Chemistry, Faculty of Science, University of Zagreb, Horvatovac 102a, Zagreb 10000, Croatia
4 Department of Biotechnological and Applied Clinical Sciences, University of L'Aquila, via Vetoio 1, L'Aquila 67100, Italy
5 National Center of Biotechnology-CSIC, Calle Darwin 3, Madrid 28049, Spain
* Correspondence:Correspondence: tondid@unimore.it (D.T.), laura.cendron@unipd.it (L.C.).
† Equally contributed to the work

Table 1. Statistics of data collection, processing and refinement. Values for the outer shell are given in parentheses.

***Pa*AmpC:3**		
**Crystallographic data**		
Crystal system		Trigonal
Space group		$P3_221$
Wavelength / Å		0.978
Unit cell parameters / Å, °		$a = b = 82.678$, $c = 123.563$
		$\alpha = \beta = 90.00$, $\gamma = 120.00$
Z	unit cell	6
	asymmetric unit	1
Resolution range / Å		46.78–1.78 (1.81–1.78)
R_{merge}		0.042 (0.567)
CC(1/2)		0.999
$\langle I / \sigma(I) \rangle$		12.6 (1.7)
Completeness / %		97.8 (92.0)
Redundancy		3.7 (3.7)
**Refinement**		
R_{work} / R_{free}		0.177/ 0.200
Protein atoms		2843
Water molecules		131
Ligand atoms		14
Phosphate ions		1
Mean B / Å^2		38.30
**R.m.s.d. from ideal value**		
Bond length / Å		0.012
Bond angles / °		1.548
**Geometry**		
Ramachandran favored / %		97.81
Ramachandran outliers / %		0.00
Rotamer outliers / %		0.68

Table 2. Hydrogen bonds between ligand molecule **3**, *Pa*AmpC residues and water molecules.

***Pa*Ampc**			Cpd **3**	
**Atom**	**Molecule**	**No.**	Atom	Distance (Å)
N	Ser	64	O14	2.88
OH	Tyr	150	O13	2.62
N	Ser	319	O14	2.86
O	W	561	O01	2.76
O	W	502	O03	2.40
O	W	567	O13	2.76
OG	Ser	64	B12	1.59*

*Denotes a covalent bond.

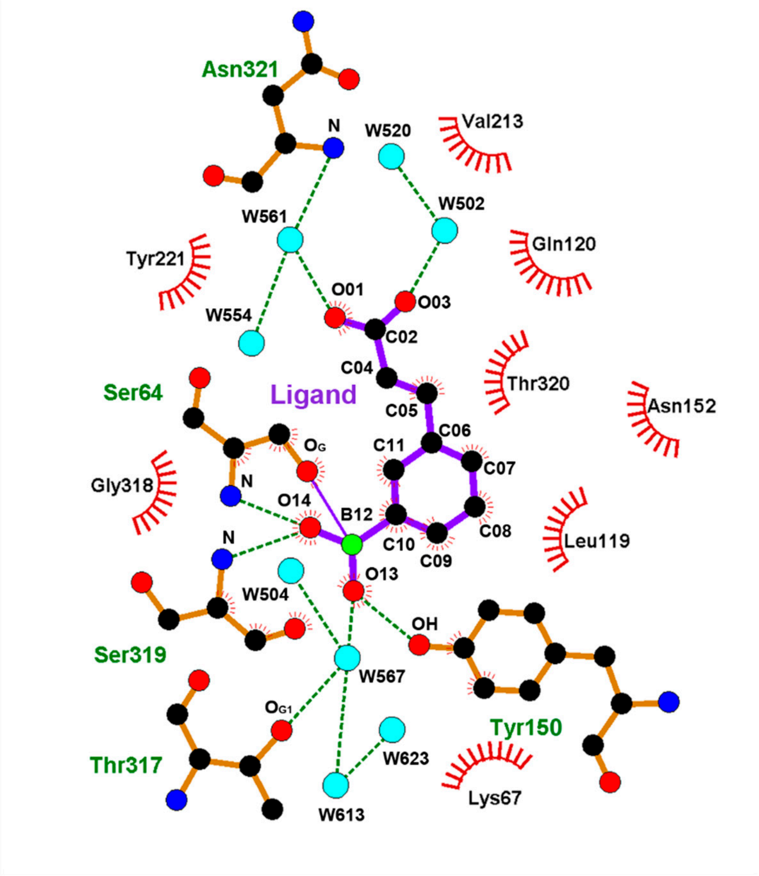

Figure S1. Key interactions between AmpC, ligand **3** and water molecules (shown as blue spheres) analyzed by Ligplus software [45]. Atoms identification codes are defined in the li-and binding site plot. Distances between the selected atoms are shown in Table S2.

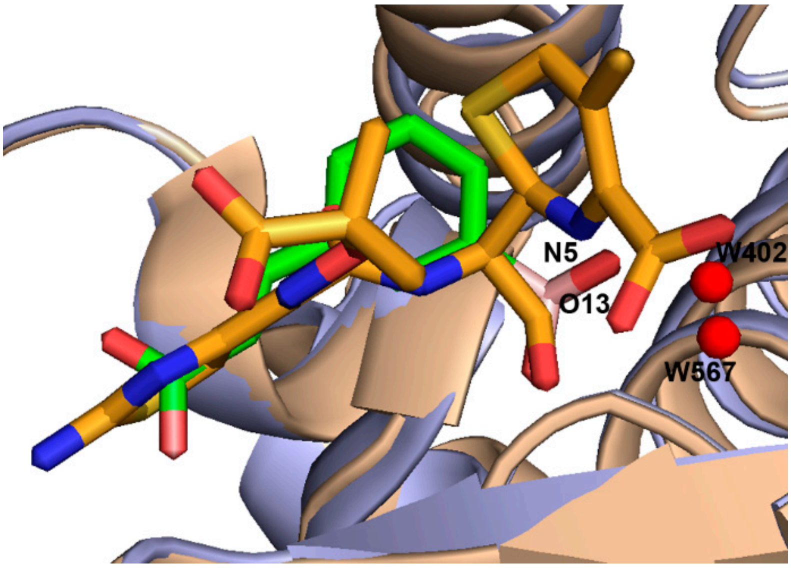

Figure S2. Overlay of the *Ec*AmpC: **ceftazidime** (wheat) and *Pa*AmpC: **3** (light blue) active sites. Ligands (ceftazidime in orange and **3** in green) are represented as sticks. De-acylating water molecules, in *Ec*AmpC:**ceftazidime** (W402) and in *Pa*Ampc:**3** (W567), are depicted as a red sphere.

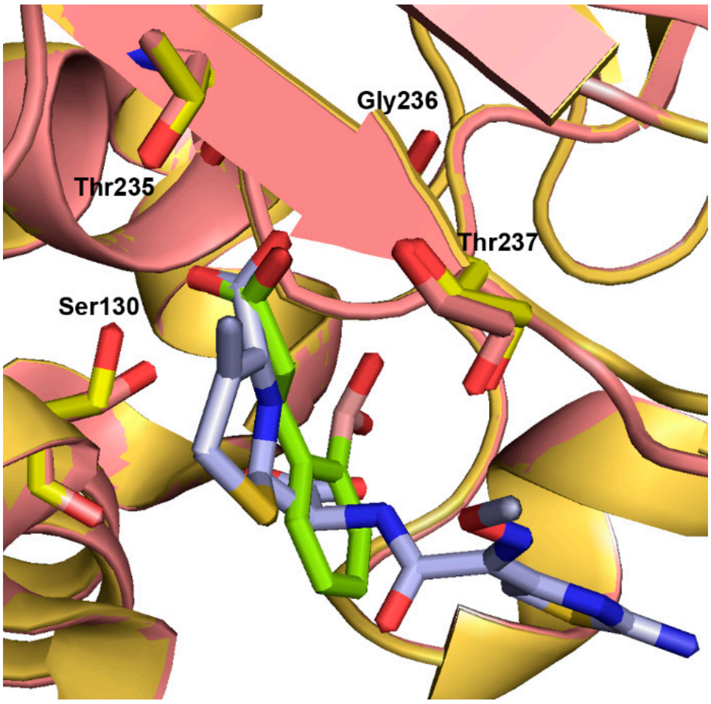

Figure S3. Overlay of the KPC-2:**cefotaxime** (salmon) and KPC-2:**2** (ye-low orange) active sites. Ligands (**cefotaxime** in light blue and **2** in lemon) are reported as sticks as well as Ser130, Thr235, Gly236 and Thr237 residues that accommodate the carboxylated side chain in both KPC-2: **ligand** structures.

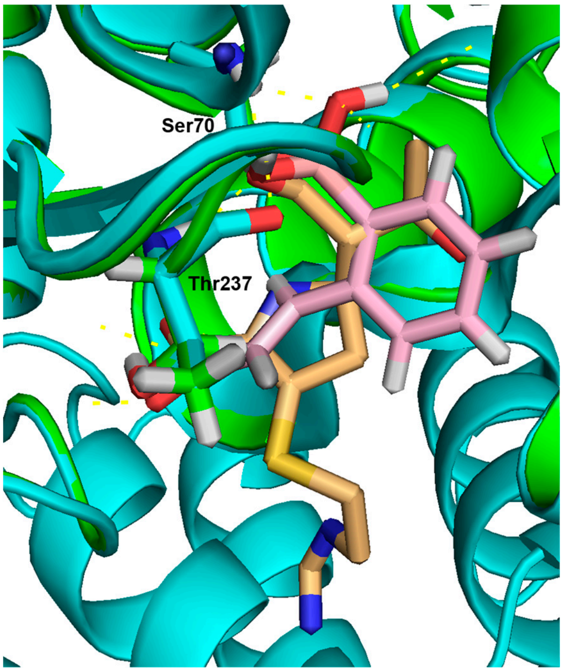

Figure S4. Overlay of GES-5: **imipenem** (turquoise) and GES-5:**2** (green) active sites. Ligands (**imipenem** in light orange and **2** in light pink) are reported as sticks, as well as Ser64 and Thr 232 involved in the key interactions for housing the oxyanion hole in both GES-5: **ligand** structures.
